# Low serum vitamin D concentrations in Spring-born dairy calves are associated with elevated peripheral leukocytes

**DOI:** 10.1038/s41598-021-98343-8

**Published:** 2021-09-23

**Authors:** Susana Flores-Villalva, Megan B. O’Brien, Cian Reid, Seán Lacey, Stephen V. Gordon, Corwin Nelson, Kieran G. Meade

**Affiliations:** 1 Animal and Bioscience Research Department, Animal and Grassland Research and Innovation Centre, Teagasc, Grange, Ireland; 2 School of Agriculture and Food Science, University College Dublin, Belfield, Dublin 4, Ireland; 3 CENID Fisiología, INIFAP, Querétaro, Mexico; 4 Department of Mathematics, Munster Technological University, Cork, Ireland; 5 UCD School of Veterinary Medicine, University College Dublin, Belfield, Dublin 4, Ireland; 6 Department of Animal Science, University of Florida, Gainesville, USA; 7 Conway Institute of Biomolecular and Biomedical Research, University College Dublin, Belfield, Dublin 4, Ireland; 8 Institute of Food and Health, University College Dublin, Belfield, Dublin 4, Ireland

**Keywords:** Innate immunity, Animal physiology

## Abstract

A role for vitamin D in the immune system is emerging from human research but data in the bovine is limited. In the current study, 48 Holstein–Friesian calves were randomly assigned to one of 4 groups designed to expose calves to divergent vitamin D levels for a 7 month period and to determine its effects on circulating immunity in young calves. Concentrations of circulating 25-hydroxyvitamin D (25OHD) was measured in serum using a commercial ELISA with validated bovine standards. Results showed that mean circulating concentrations of 25OHD at birth was 7.64 ± 3.21 ng/ml indicating vitamin D deficiency. Neither the injection of Vit D_3_ at birth nor the elevated levels in milk replacer yield discernible changes to pre-weaning circulating concentration of 25OHD. No calf reached the recommended level of vitamin D immune sufficiencyof 30 ng/ml of 25OHD until at least 3 months of age (T4). Increasing dietary Vit D_3_ via ration in the post-weaning period significantly elevated 25OHD concentrations in serum in VitD-In calves. Maximal levels of circulating 25OHD were achieved in VitD-Out calves, reaching 60.86 ± 7.32 ng/ml at 5 months of age (T7). Greatest divergence in haematology profile was observed between Ctl-In vs VitD-In groups with Ctl-In calves showing an elevated count of neutrophils, eosinophils, and basophils associated with reduced 25OHD concentrations. Neither IL-8 expression nor ROS production in serum were significantly different between calves with high and low 25OHD, indicating that other vitamin D-dependent mechanisms may contribute to the divergent circulating cellular profiles observed. This novel data on the vitamin D status of neonatal calves identifies a significant window of vitamin D insufficiency which is associated with significant differences in circulating immune cell profiles. Vitamin D insufficiency may therefore exacerbate pre-weaning disease susceptibility, and further work in now warranted.

## Introduction

Vitamin D (Vit D) is the collective term used to describe a group of closely related fat-soluble steroids. Their main biological function is to maintain serum calcium and phosphorous concentrations within the normal range by enhancing the efficiency of the small intestine to absorb these minerals from the diet. In cattle, the most important compounds in this group are vitamin D_2_ (Vit D_2_) and vitamin D_3_ (Vit D_3_). Vit D_2_ (ergocalciferol) is obtained from the roughage used for cattle feed (e.g., hay and silage) and Vit D_3_ (cholecalciferol) is synthesized in the skin during exposure to sunlight^1^, and it is also supplied as a synthetic supplement in feed^2^. Although both molecules, are metabolized in the same way, Vit D_2_ is less physiologically effective and less efficient at ensuring a sufficient blood levels of Vit D in cattle^3^. Vit D is biologically inert and must be activated by two sequential hydroxylations. The first hydroxylation occurs in the liver to produce 25-hydroxyvitamin D (25OHD), the main circulating form used to determine Vit D status; this is then converted to the active metabolite 1,25-dihydroxyvitamin D (1,25OHD) in the kidney but also in many peripheral tissues and cells from the immune system such as macrophages and monocytes^4^.

25OHD status results from multiple non-exclusive factors including season, UVB exposure, nutrition, age, sex, and productive stage^5^. Although the optimal concentration of circulating 25OHD is still a matter of debate, a value of 30 ng/ml is recommended^6^. In the EU, Vit D_3_ is the only authorised source of supplemental Vit D for cattle, with the maximum permitted levels set at 10,000 IU/kg for milk replacer and 4000 IU/kg in feed^7^. However, information on the vitamin D status of cattle in general, and calves in particular is limited. Data is available for US systems^8,9^, but different genetics and systems of feeding management means these values are not directly transferrable to calves under less intensive production systems and in different geographical locations. In Ireland, milk replacer usually contains 6000 IU/kg of Vit D_3_, and in previous studies, we observed a high prevalence of low 25OHD serum concentration in calves during the first 5 months of life. Moreover, seasonal Vit D profile was negatively correlated with the expression of the pro-inflammatory chemokine interleukin-8 (IL-8) suggesting relevant immune consequences^10^.

The immune system in calves develops gradually from conception to maturity at approximately 6 months after birth^11^. During this time, calves are particularly vulnerable to infection with respiratory and enteric bacteria and viruses (respiratory syncytial virus, BVD, herpesvirus, *E. coli,* rotavirus, *Salmonella*). However, these infections are often secondary and opportunistic, resulting from animals with a compromised or underdeveloped immune system. Adequate Vit D is now viewed as vital for optimal health with research showing important associations between 25OHD blood concentration and immune function. Studies performed predominantly in humans have shown important immunomodulatory and antimicrobial effects of Vit D on innate cells including macrophages and neutrophils^4^. More recent studies have also associated Vit D with microbiome development, indicating another potential route toward immune system involvement^12^. Specifically in cattle, it has been hypothesised that low circulating levels of 25OHD may be associated with respiratory disease^13^. It is therefore probable that sub-optimal Vit D status could have negative consequences for optimal calf immune system development and consequently affect disease susceptibility. Thus, in this study we aimed to develop a model to drive divergent Vit D status under the current European supplementation guidelines and to investigate how variation in the circulating concentration of 25OHD affected the immune cell profiles in dairy calves.

## Results

### Vitamin D deficiency in calves at birth extends until 3 months of age and is not addressed by supplemented Vit D_3_ in milk replacer or ration pre-weaning

**Figure 1:**
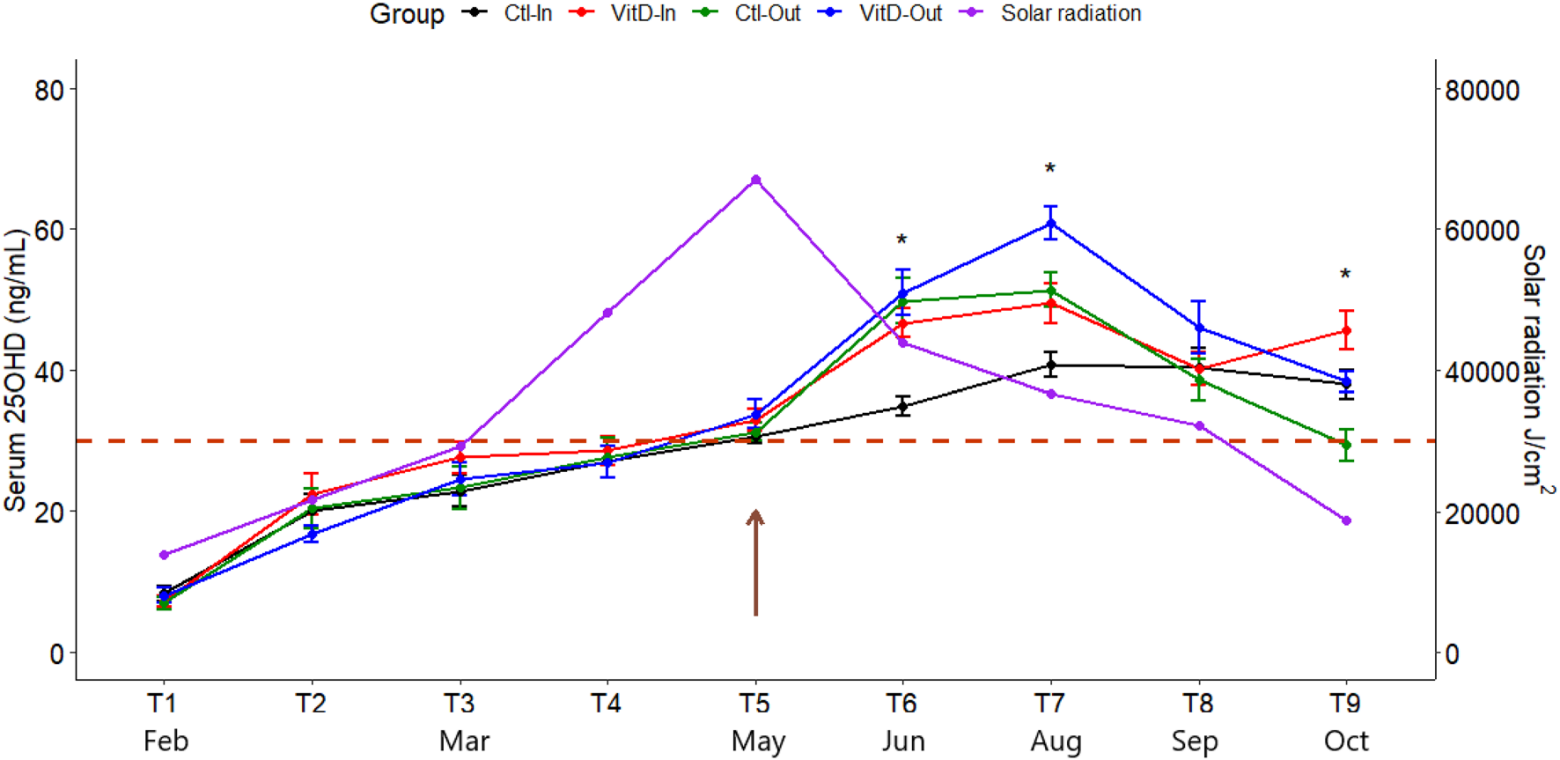
Vitamin D_3_ supplementation and sunlight exposure increases 25OHD circulating levels in calves. Ctl-In (n = 11) and VitD-In (n = 12) groups were kept indoors and fed with 6000 IU/kg in MR + 2000 IU/kg of Vit D_3_ in ration; or 10,000 IU/kg in MR + 4000 IU/kg of Vit D_3_ in ration, respectively. Whereas Ctl-Out (n = 11) and VitD-Out (n = 10) groups were move outdoors after weaning and fed with 6000 IU/kg in MR + 2000 IU/kg of Vit D_3_ in ration; or 10,000 IU/kg in MR + 4000 IU/kg of Vit D_3_ in ration, respectively. All calves received a single time injection of 50,000 IU of Vit D_3_ at T1, except Ctl-In, which received a vehicle injection with ethanol. Serum samples were taken at the beginning of the trial (T1), after 15 days (T2) and at 30, 70, 90, 130, 160, 200 and 230 days after T1 (T3-T9). Data represent mean and standard error of serum 25OHD profile in calves for each time point within each group. The main effect of sunlight and treatment was significant (*p* < 0.05) at T6, T7 and T9. Red dotted line shows the recommended 25OHD serum level of 30 ng/ml. Brown arrow shows the time where Ctl-Out and VitD-Out groups were moved outside. Right Y-axis represent the solar radiation during the time points where 25OHD concentrations were analysed.

In this study we analysed the effects of Vit D_3_ supplementation on the neonatal Vit D deficiency and its impact on the bovine immune response in early life. The average circulating concentrations of 25OHD across all calves at birth (T1) was 7.64 ± 3.21 ng/ml, indicating Vit D deficiency. Despite a sub-dermal injection of Vit D_3_ (50,000 IU) at birth and inclusion of supplementary Vit D_3_ in milk replacer (6000 IU/kg in Ctl groups and 10,000 IU/kg in Vit D groups), a change in serum 25OHD concentration at 15 days post-Vit D_3_ injection (T2) was not detected (*p* > 0.05) (Fig. 1).

Insufficiency continued in all treatment groups until calves were on average 3 months of age (T4) (Fig. 1). Average values for T2 were 19.86 ± 7.84 ng/ml, T3 were 24.61 ± 8.22 ng/ml and for T4 were 27.55 ± 7.74 ng/ml. During this period, calves were on an exclusively milk diet and no statistically significant differences due to Vit D_3_ treatment was evident pre-weaning (*p* > 0.05) (Table 1).

**Table 1:** Serum 25OHD concentrations (ng/ml) within each group for all time points assessed.

Time	Ctl-In (n = 11)		VitD-In (n = 12)		Ctl-Out (n = 11)		VitD-Out (n = 10)		Overall mean*	Overall SD*	*p*-value^3^		
	Mean	SD	Mean	SD	Mean	SD	Mean	SD			Treatment^1^	Sunlight^1^	Treatment × Sunlight^2^
T1	8.34	3.619	7.24	2.769	6.94	3.054	8.04	3.382	7.64	3.206	0.908	0.6541	0.217
T2	20.00	8.027	22.34	10.222	20.42	9.406	16.68	3.699	19.86	7.839	0.533	0.578	0.094
T3	22.90	7.654	27.62	7.72	23.37	10.059	24.55	7.441	24.61	8.219	0.32	0.758	0.382
T4	27.03	7.584	28.58	6.846	27.62	9.279	26.96	7.253	27.55	7.741	0.948	0.896	0.404
T5	30.70	3.421	32.88	5.465	31.21	4.194	33.83	6.589	32.16	4.917	0.195	0.436	0.82
T6	34.96	4.412	46.71	6.769	49.81	10.554	50.99	9.921	NA	NA	0.021	0.000411	0.033
T7	40.79	5.765	49.46	9.945	51.36	7.921	60.86	7.318	NA	NA	0.00075	0.0000891	0.879
T8	40.42	8.798	40.13	8.217	38.66	9.592	46.04	11.654	NA	NA	0.198	0.597	0.159
T9	37.98	6.788	45.71	9.517	29.38	7.577	38.33	4.821	NA	NA	0.001	0.002	0.86

*When no statistical difference was observed between treatments, an overall mean and SD was obtained for all groups, *NA* not applicable, *n* number of animals.

^1^Main effects of treatment and sunlight.

^2^Interaction between treatment and sunlight.

^3^Interaction among treatment, sunlight and time was significant (*p* < 0.05).

### Greatest serum 25OHD concentrations are achieved by a combination of dietary Vit D_3_ supplementation and sunlight exposure

After weaning (T5, Fig. 1), one control group (Ctl-Out) of calves (maintained on the industry standard concentrate containing 2000 IU/kg of Vit D_3_) and one treatment (VitD-Out) group (received Vit D_3_ supplementation to 4000 IU/kg of Vit D_3_) were moved outdoors to grass, while groups Ctl-In and VitD-In were kept indoors. Circulating concentrations of 25OHD increased over time in VitD-In, Ctl-Out and VitD-Out groups whereas concentrations of 25OHD in the Ctl-In group remained the lowest after weaning (T5–T8). The slight increase across time points in Ctl-In is likely due to incidental sun exposure indoors (Fig. 1). The increase in circulating 25OHD in Ctl-Out and VitD-Out groups coincided with the highest solar radiation recorded for the year during May and started to decline after peak sun exposure at T8 (Sept) (Fig. 1).

**Figure 2:**
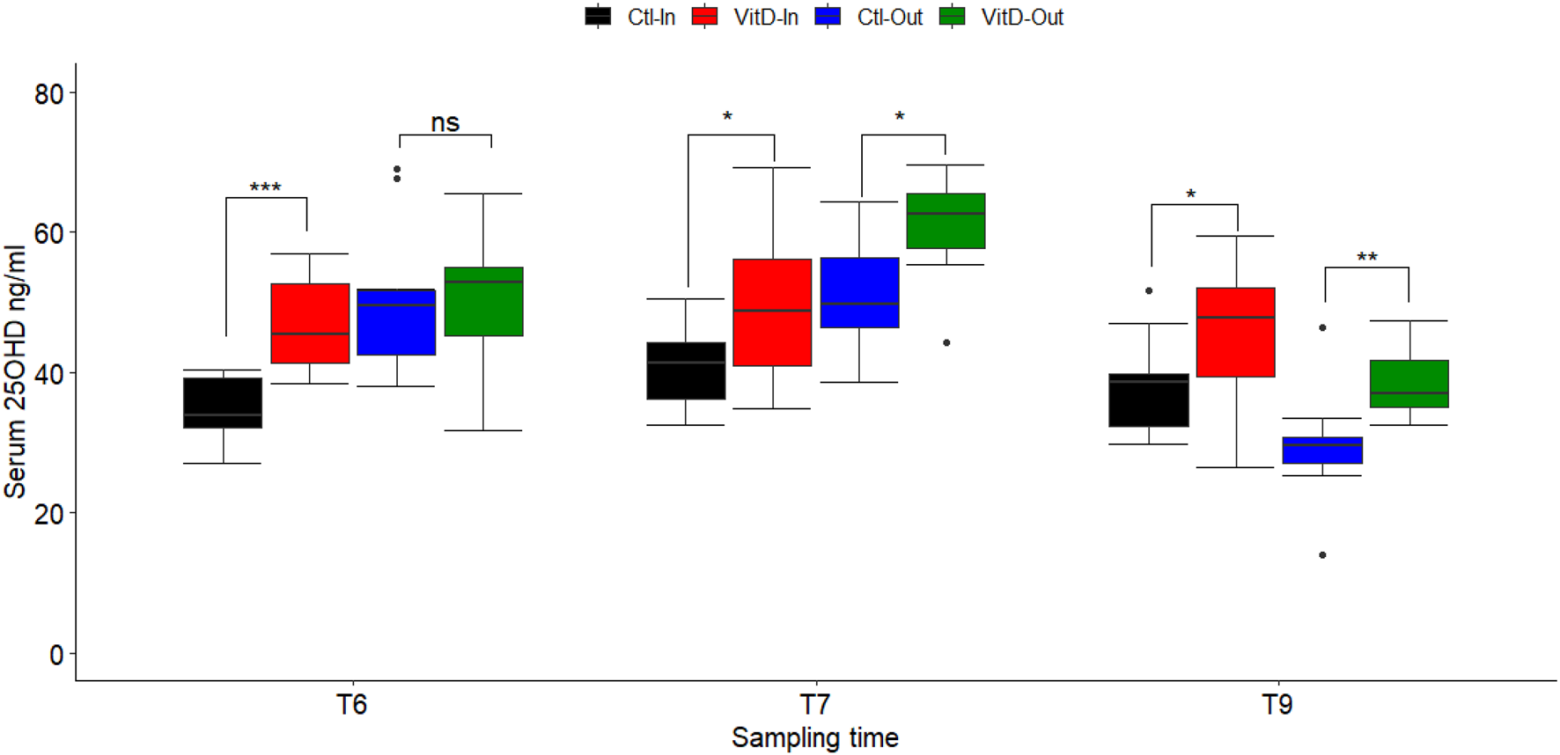
Maximum 25OHD levels are achieved by dietary VitD_3_ supplementation and sunlight exposure. Boxplots of serum 25OHD levels in calves within each group at time T6, T7 and T9. Differences between groups were calculated by a 2 × 2 factorial design as described in the material and methods section. Dots represent outlier values. *p < 0.05, **p < 0.01, ***p < 0.001, *ns* not significant. Ctl-In (n = 11), VitD-In (n = 12), Ctl-Out (n = 11) and VitD-Out (n = 10).

The main effect of treatment and sunlight was significant in all groups at T6, T7 and T9 (*p* < 0.05) (Table 1). A significant interaction between treatment and sunlight was observed at T6 (*p* < 0.05), although no significant interaction was detected at T7 or T9 (*p* > 0.05) (Table 1). At T6 no statistical difference (*p* > 0.05) in the 25OHD concentration was observed between Ctl-Out and VitD-Out groups. In contrast, a significant divergence in 25OHD levels was observed between Ctl-In and Vit-In groups (*p* < 0.05) (Fig. 2). The highest 25OHD concentration was observed at T7 in VitD-Out group, with an average 25OHD concentration of 60.86 ± 7.32 ng/ml in comparison with 51.36 ± 7.92 ng/ml in Ctl-Out group (Table 1). As the increase was lower in VitD-In than in the outdoor supplemented group (VitD-Out), results show evidence for an additive effect between dietary supplementation and sun exposure (Fig. 2).

### Low circulating levels of 25OHD are associated with a higher number of granulocytes in circulation

**Figure 3:**
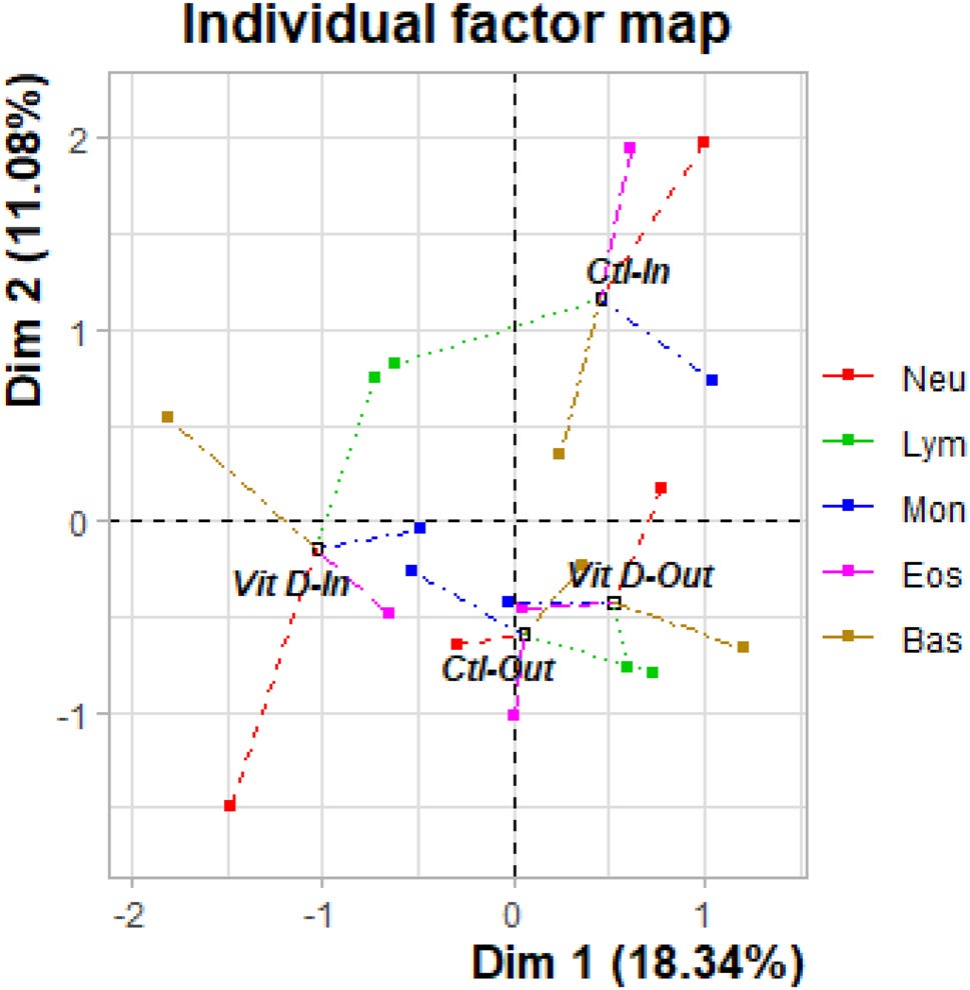
Changes in overall cell composition across time in all groups. The haematology profile for all time points was analysed by MFA as described in material and methods section. The individual factor map shows each group represented at the barycentre of their individuals. Individuals are projected on the side of the group of variables for which they have a high value, and opposite those variables for which they have low value. Lym, Bas and Mon are represented in Dim1, whereas Neu, Eos are represented in Dim2. Ctl-In (n = 11), VitD-In (n = 12), Ctl-Out (n = 12) and VitD-Out (n = 11).

To analyze the potential relationship between circulating levels of 25OHD and hematological profile, a multiple factorial analysis (MFA) was performed using values for predominant cell profiles of neutrophils, eosinophils, basophils, lymphocytes, and monocytes (Neu, Eos, Bas, Lym, and Mon) on all calves across all time points (Fig. 3). Overall, the cell profile of Ctl-Out and VitD-Out groups was similar; the barycentre (overall mean) of these groups are situated close together compared to indoor groups. In contrast, Ctl-In and VitD-In groups show a distance relative to the outdoor groups, indicating a change in overall circulating cell composition across time; therefore, its within inertia values for Dim2 were higher (Suppl. Table 1). The most divergent group was Ctl-In, which separate away from the other groups along dimension 2, which explains 11.22% of the phenotypic variation (Fig. 3). Finally, the MFA shows that the cells making the most significant contribution to the divergence between Ctl-In and VitD-In are Neu, Eos, and Bas indicated by the greater distance between the partial points for each group (Fig. 3).

**Figure 4:**
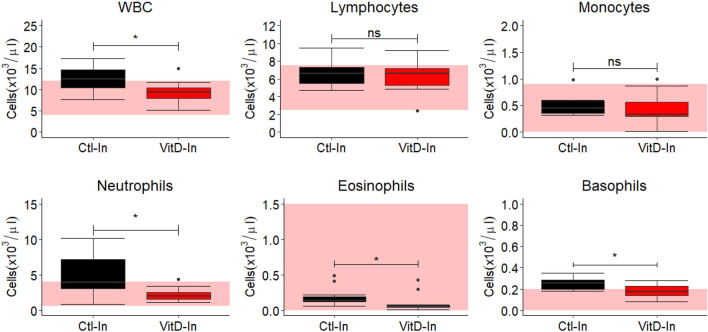
Collective differences in the cell profile between indoor groups at T6. Data shows the boxplot of WBC (white blood cells), neutrophils, eosinophils, basophils, lymphocytes, and monocytes of Ctl-In (n = 11) and VitD-In (n = 12) groups at T6**.** Collective differences between groups were analysed by MANOVA as described in material and methods section. Pink area shows references values according to Merck Veterinary Manual. Dots represent outlier values. *p < 0.05, *ns* not significant.

Multivariate statistical analysis of covariance within each cell type (Suppl. Figure 2) shows that the principal significant differences in Neu, Eos, and Bas occurred at T6 (*p* < 0.05). This time point (T6) was where a high divergence in the blood 25OHD concentration between Ctl-In and VitD-In groups was observed (Fig. 2) and the analysis of the cell profile at T6 shows that calves from Ctl-In group had higher number of Neu, Eos, and Bas in comparison with animals from VitD-In (*p* < 0.05) (Fig. 4). Notably, the cell counts are increased relative to reference values for cattle which is indicative of neutrophilia. These results suggest that elevated 25OHD is associated with reduced numbers of circulating leukocytes.

### Divergence in circulating 25OHD does not induce significant differences in IL-8 expression or ROS production

**Figure 5:**
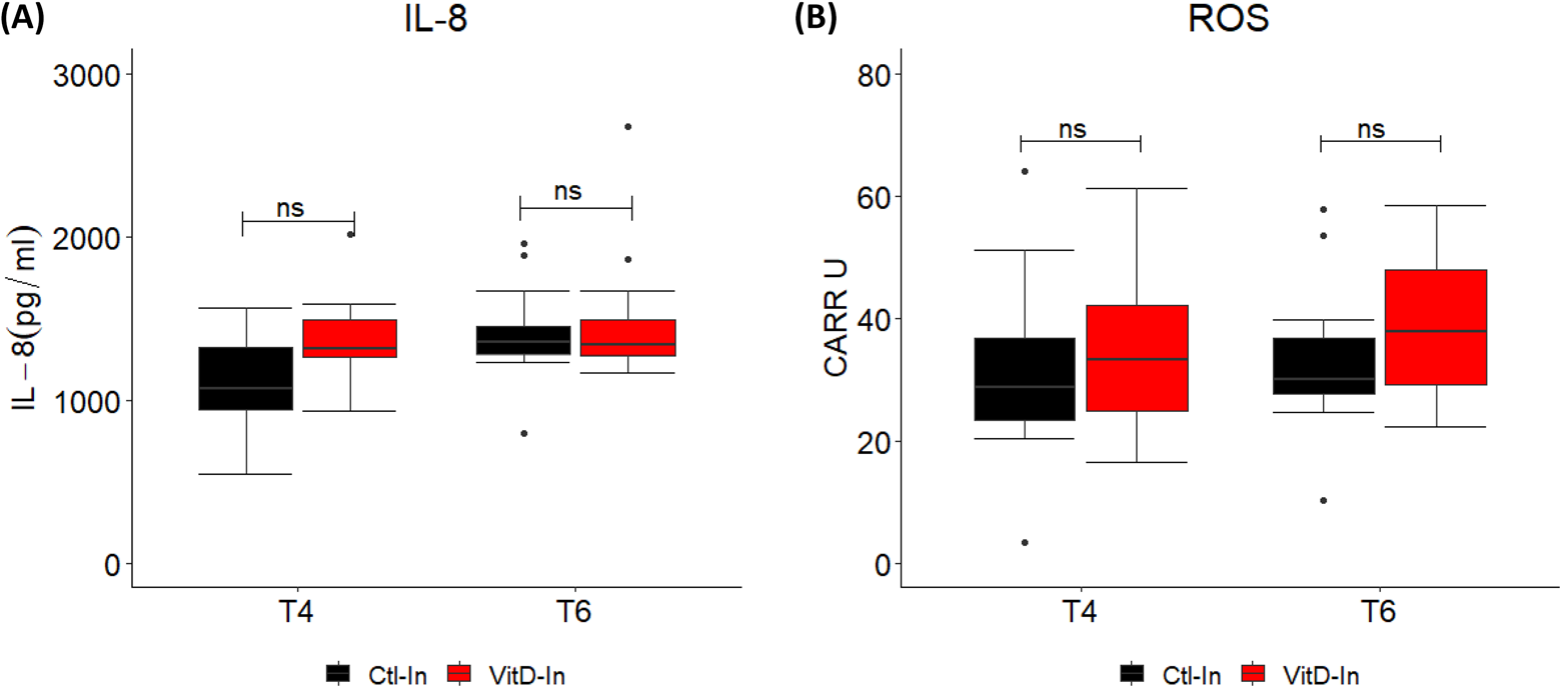
Serum concentration of IL-8 and ROS in calves from indoor groups. Data shows the boxplot of serum concentration of IL-8 and ROS at T4 and T6 in calves from Ctl-In (n = 11) and VitD-In (n = 12) groups**. **(**A**) IL-8 protein levels were measured by ELISA. (**B**) ROS were measured in serum using the d-ROM test. Data was analysed by ANOVA test. Dots represent outlier values. *ns* not significant.

To investigate potential mechanisms by which elevated circulating 25OHD may suppress numbers of circulating immune leukocytes, IL-8 expression and ROS production in serum were evaluated in the most 25OHD divergent groups (Ctl-In and VitD-In). The serum concentrations were evaluated at time point 4 (T4) where the Vit D status was similar between calves, and at time point 6 (T6) the most differing time point (Fig. 1). We did not observe a statistically significant difference (*p* > 0.05) in IL-8 expression levels in calves from Ctl-In and VitD-In groups at any of the time points evaluated (Fig. 5A). Similarly, we did not observe a statistical difference (*p* > 0.05) in the ROS production between groups at any time point, although there is a trend of higher ROS levels in VitD-In animals (Fig. 5B).

## Discussion

Neonatal mortality and morbidity remains a significant issue for the dairy industry^14^. Nutritional strategies to support optimal immune system development may hold significant promise to reduce these losses, particularly in artificially reared dairy calves. Our work has recently identified that Spring-born dairy calves have a low level of circulating vitamin D in serum^10^. However, the consequences of such concentrations for the development of the immune system have not been clearly defined. The current definition of Vit D requirement in cattle diets is based on concentrations required to maintain the bone and mineral homeostasis^15^; however, the optimal 25OHD concentration for immunity has not been conclusively determined. Given the similarities in Vit D metabolism between humans and cattle, Vit D requirements for humans are adopted in cattle; thus, vitamin D deficiency is defined as circulating 25OHD concentrations below 20 ng/ml and insufficiency as levels between 20–29 ng/ml. Therefore, a 25OHD blood level above 30 ng/ml is deemed to be the target to improve the health and resistance to infections^6,16^. Furthermore, as levels of Vit D supplementation is tightly regulated within the EU, it is critical to understand if circulating levels of Vit D in calves under current dairy production systems are sufficient.

In this study, calves at birth were Vit D deficient, with 25OHD levels below 10 ng/ml. These blood levels did not reach the optimal threshold until animals were on average 3 months of age, thereby exposing them to a potentially prolonged period of disease susceptibility. The 25OHD levels reported here are significantly lower than previous reports in US dairy studies, where calves had 25OHD levels within the range of 15–40 ng/ml at birth^17,18^. Our results show a failure of supplemental injection of 50,000 IU of Vit D_3_ to impact circulating levels of 25OHD, despite sub-dermal injections having previously been shown to effectively improve Vit D concentrations in calves at birth^17,18^. This is likely explained by the divergent concentrations used as previous work used an initial injection of 150,000 IU of Vit D_3_ followed by 5000 IU daily, which resulted in an increase of 25OHD levels from 30 ng/ml at birth to near 100 ng/ml after 14 days in calves raised indoors^17^. In addition, in our study, supplemented milk replacer, at maximal levels did not significantly influence circulating 25OHD levels. Nevertheless, the neonatal Vit D concentrations in our calves were significantly lower than in this previous study, suggesting that a concentration of more than 150,000 IU of Vit D_3_ may be required for Irish calves to reach a target of 30 ng/ml 25OHD serum levels. Parenteral administration of Vit D_3_ provides a better degree of precision than oral supplementation, particularly under situations of group feeding. In fact, Nonnecke et al*.* developed a model of Vit D_3_ supplementation by a subcutaneous injection of 25OHD over a period of 28 days, achieving higher 25OHD serum concentrations and less inter-animal variability^19^. However, there are no long-term studies of parenteral administration of Vit D_3_ in cattle and furthermore the assessment of parenteral Vit D_3_ injections at time critical points, such as weaning, is needed.

At weaning, the average 25OHD concentration was close to the optimal level of the desired 30 ng/ml threshold in all calf groups. However, it could be considered low according to the concentrations observed in US dairy calves raised indoors and fed with a similar Vit D_3_ rate (6600 IU/Kg of DM) with a mean serum 25OHD concentration near 60 ng/ml at 6 weeks of age^18^. Thus, calves under more intensive dairy systems have considerably elevated Vit D concentrations than pasture-based calves born in Spring. One factor that could contribute to the divergent circulating 25OHD concentrations in calves between studies is the concentration of Vit D in the dam. Weiss et al., showed a positive correlation with the serum 25OHD concentrations in calves at birth with concentrations of 25OHD in the serum of their dams^20^. 25OHD concentrations from dams were not available for this study; however, all the calves were purchased from a single farm and so variation in individual cows’ intake would not be expected to contribute significantly to these results. Nonetheless, our results raise important questions regarding the vitamin D status of pasture-based dairy cows, and indeed suggests a potential strategy whereby dietary supplementation of the dam could be a possible future solution to vitamin D deficiency in Spring-born dairy calves.

Dietary supplementation in the post-weaning period did successfully increase 25OHD concentrations in this study. Peak 25OHD concentrations were achieved using a combination approach of dietary supplementation and sun exposure which occurred in the month following peak solar exposure. The maximum 25OHD blood levels were observed at time point 7, with average 25OHD concentration of 60.86 ± 7.32 ng/ml in Vit D supplemented calves exposed to sunlight; whereas Vit D supplemented calves kept in the shade had 49.46 ± 9.95 ng/ml. This is similar to levels observed in 5–6 month old beef calves grazing on the summer in central US with a mean serum 25OHD concentration between 50 to 60 ng/ml with minimal Vit D_3_ supplementation (~ 80 to 190 IU/kg of DM)^21^. Nevertheless, 25OHD blood levels up to 100 ng/ml had been reported in cows grazing at 30° N with an estimated intake of 2800 IU of Vit D_3_ per day^8^. Although there are no data regarding the optimal range of 25OHD levels, evaluation of the macrophage responses in vitro had shown a linear benefit to increasing 25OHD concentrations up to 100 ng/ml^22^.

Multiple factors are known to regulate Vit D_3_ skin synthesis, including latitude, altitude, and time of exposition to sunlight^23^. As in humans, seasonal variation in the Vit D status on grazing calves (with minimal or null Vit D_3_ supplementation) has been reported, with low 25OHD concentration in winter and early spring^21,24^. Thus, at northern latitudes (> 40°N) cutaneous production of Vit D_3_ is not sustained throughout the year. The above is supported by our results which suggest that Vit D_3_ skin synthesis in pasture-based calves raised in Ireland (53°N) is limited from autumn to early spring. Furthermore, our results show that calves without access to sunlight require a Vit D_3_ supplementation of at least 4000 IU/Kg to maintain 25OHD concentration similar to that achieved under sunlight exposure during summer months.

In this study, a clear effect of divergent vitamin D profile on circulating immune cell populations was demonstrated. Specifically, we detected that calves with low 25OHD concentration had an increase in the overall circulating cell composition across time in comparison to calves with higher 25OHD levels. Our results show that the main changes in leukocytes were significant in neutrophils, eosinophils, and basophils. Previous short-term Vit D_3_ supplementation studies for 30 days did not report changes in the leukocyte populations^25–27^ and no long-term Vit D_3_ supplementation studies had previously assessed the effects on the immune response in cattle.

Haematological profiles in dairy calves are limited^28,29^, and research had shown that the use of adult cows’ reference interval values is inaccurate for neonatal calves in the first 5–8 weeks of life^30^. However, in this study all the average values obtained after this period were within the values reported for healthy beef and dairy calves with differences only observed in calves with low 25OHD levels^28,31^. These immune cell changes could have important consequences for disease susceptibility. Neutrophilia is associated with stress and inflammatory process such as infectious diseases^32^. However, all the calves were clinically healthy when the increase in the immune cells was evident. IL-8 is a potent chemotactic agent for neutrophil recruitment and inflammation, and previously, we observed an inverse relationship between circulating 25OHD levels and IL-8 expression in calves with two different IL-8 haplotypes^10^. In this study, IL-8 expression was not associated with the changes in 25OHD levels. Therefore, our results suggest that although IL-8 genotype shapes Vit D responses, other molecular factors are associated with the Vit D status in cattle. In fact, a recent genome-wide association study in African calves had found serum 25OHD concentration to be under polygenic control^33^. Therefore, environmental and host factors can contribute to changes in the Vit D status of cattle. Another potential mechanism underlying the neutrophilia reported here is an increased release of cells from the bone marrow. The exit of mature neutrophils from the bone marrow is tightly regulated by a cytokine network, which includes IL-17^34^. IL-17 production is known to be modulated by vitamin D^35^, and Vit D restriction results in the overexpression of IL-17^36^, which identifies potentially productive avenues for future research. Vitamin D has widespread effects on cell differentiation, proliferation and cytokine modulation^37^, and identification of the precise relationship between these and related variables will be the focus of future work.

Reactive oxygen and reactive nitrogen species (ROS/RNS) are key players in cellular signalling and regulation of oxidative stress. Neutrophils are known to produce a large amount of ROS and RNS, and although it is a highly regulated process, under certain circumstances, such as chronic inflammatory diseases, neutrophils can be triggered to release ROS/RNS causing damage to host tissues^38^. Vit D is one of the key controllers of systemic inflammation and oxidative stress, and its deficiency may contribute to the dysregulation of ROS signalling pathways^39^. We measured the reactive oxidative metabolites (ROMs) in serum as a reference for ROS production^40^. However, we did not observe differences in ROS production between animals with divergent 25OHD concentrations. The lack of difference in our results could be due to the lower ROS production in calves in comparison with adult cattle, with previously published reports of ROS concentrations 64% lower in calves as compared to adults^41^. Furthermore, while the serum analysis of ROMs has been validated in dairy cattle for the analysis of free radicals of oxygen^40^, ROS and RNS represent a broad range of molecules with distinctive properties which are challenging to detect^42^. Therefore, the assessment of the oxidative stress index (based on the ratio between ROS and serum antioxidant capacity) has been suggested as a more accurate approach to determine the oxidative status ^43^. Thus, future studies should assess the production of antioxidant factors to better define the oxidative status in calves with divergent 25OHD levels. Previous work showed that Vit D modulates nitric oxygen production via NOS2 gene expression in cattle^44,45^. Our results show a trend for higher ROS levels in calves with high 25OHD levels. Merriman et al. showed that 25OHD induced elevated NOS2 gene expression, and its expression was upregulated upon LPS stimulation in macrophages and neutrophils from milk^45^. Whether high ROS/RNS levels under non-inflammatory conditions provides resistance to infections remains to be determined.

Finally, an alternative Vit D pathway by CYP11A1 and an extensive network of Vit D metabolites has been described^46^. The metabolites of this novel pathway have been identified in human epidermis, serum, placenta, and pig adrenal glands. However, its physiologic relevance in humans and cattle is unknown^47^. Furthermore, their dynamics after sun exposure or Vit D supplementation are yet to be explored^48^. Therefore, a comprehensive study of the Vit D metabolome beyond 25OHD is necessary to gain a more comprehensive understanding of the role of Vit D in health and disease^49^.

## Conclusion

Studies on the Vit D profile on young calves are sparse and to our knowledge this is the first study of a long term Vit D_3_ supplementation in calves from birth to 7 months of age. Our results identify Vit D deficiency in Spring-born dairy calves which significantly perturbs the cellular immune response. Deficiency of Vit D could have important implications for calf health, not only on immune system development and the microbiome^50^ but also in terms of bone development^51^. Sub-optimal immune system development in early life will inevitably contribute to a failure to thrive and potentially a lifetime of disease susceptibility as well as an overdependence on antibiotic usage^14^. We have also demonstrated that Vit D_3_ supplementation within the current EU guidelines is not sufficient to improve the Vit D status of calves during the pre-weaning period. Moreover, animals with low Vit D status display a divergent cell profile, although the implications for health and disease susceptibility remain to be determined. The antimicrobial and immunoregulatory role of Vit D may offer a low-cost and effective supplement to boost natural disease resistance in cattle^52^ and this offers an intriguing area for further investigation.

## Methods

### Ethical statement

All experimental procedures were approved by the Teagasc Ethics Committee (TAEC237-2019) and were conducted under the experimental license (AE19132/P105) from the Health Products Regulatory Authority in accordance with the cruelty to Animals Act (Ireland 1876) and the European Community Directive 2010/63/EU. Reporting in the manuscript follows the recommendations in the ARRIVE guidelines.

### Animals and housing

The study was conducted at Teagasc, Grange in Ireland (53°N) between February and October 2020. Forty-eight Holstein–Friesian bull calves from a single farm, born between February and March were enrolled in the experiment. Calves were removed from the dam and fed 6 L of colostrum within 4 h of birth and were transported to the research farm within 24–48 h. Transfer of adequate passive immunity was assessed in serum by optical refractometry, calves had a mean Brix value of 9%^53^. All calves were group housed and fed in buckets with milk replacer (MR). Calves were fed with 3 L of MR from 0 to 14 days, 6 L from 15 to 60 days of age, and then 3 L from 60 to 70 days. Ad libitum access to starter pellets and water was provided via bucket. Weaning occurred at 70 days of age on average, then a commercial pellet was offered once a day. Outdoors (Ctl-Out, VitD-Out) groups were moved to outside areas after weaning and were rotationally grazed from May to October. Indoor (Ctl-In, VitD-In) groups were kept in confinement during the duration of the trial and were offered hay and silage ad libitum.

### Experimental design and treatments

The experiment was a randomized complete block design with a two-by-two factorial arrangement of treatments, with calves randomly assigned to one of 4 treatments. Treatments were arranged as a factorial with two sunlight access (indoors = In, or outdoors = Out) and two vitamin D_3_ diets (Ctl and VitD). Therefore, the four treatments were: Ctl-In: Indoors and 6000 IU/kg in MR + 2000 IU/kg of Vit D_3_ in ration; VitD-In: Indoors and 10,000 IU/kg in MR + 4000 IU/kg of Vit D_3_ in ration; Ctl-Out: Outdoors and 6000 IU/kg in MR + 2000 IU/kg of Vit D_3_ in ration; VitD-Out: Outdoors and 10,000 IU/kg in MR + 4000 IU/kg of Vit D_3_ in ration. A single time injection of 50,000 IU of Vit D_3_ was administered subcutaneously to all calves, except Ctl-In, which received a vehicle injection with ethanol.

A commercial milk replacer with 6000 IU/kg and a commercial pellet with 2000 IU/kg were used for the Ctl diets. For the Vit D_3_ diets the MR and pellet were supplemented with Vit D_3_ to achieve 10,000 IU/kg and 4000 IU/kg, respectively. The Vit D_3_ was prepared from dry powder concentrate (Rovimix D3 500, DSM Nutritional Products) containing 500,000 IU per gram of Vit D_3_ by adding 0.5 g of the concentrate to distilled water. The supplements were prepared fresh weekly and stored at 4 °C. Supplements were added once daily to the MR, and top dressed on the pellets after weaning. The ration was provided once a day at a rate of 1–4 kg per day from 70 to 220 days of average age. The feed was offered to calves at approximately 0800 hours to ensure consumption of the concentrate.

### Haematology, serum 25-hydroxyvitamin D and IL-8 ELISA

Blood samples were collected via the jugular vein into vacutainer tubes (Becton Dickinson). A sample of 6 ml blood collected in an EDTA tube was used for haematology analysis using the ADVIA 2120 haematology system. Another 10 ml serum separator tube was utilized for serum collection. Tubes were centrifuged at 2500×*g* for 15 min for serum separation within 1 h of sample collection. Serum samples were transferred into microtubes and stored frozen at − 20 °C. Sampling time points T1–T9 are defined as: T1 = beginning of the trial; T2 = 15 days after T1; T3 = 30 days after T1; T4 = 70 days after T1; T5 = 90 days after T1; T6 = 130 days after T1; T7 = 160 days after T1; T8 = 200 days after T1; and T9 = 230 days after T1.

The serum samples were analysed for concentrations of total 25OHD using an ELISA (Human 25-OH Vitamin D ELISA, Eagle Biosciences, Nashua, NH) and was carried out as per the manufacturer’s instructions using bovine standards, prepared as previously described^8^. The bovine IL-8 ELISA used to measure IL-8 concentration was carried out as previously described by Cronin et al.^54^.

### Determination of reactive oxygen metabolites

The reactive oxygen metabolites (ROM) were quantified with the standardized d-ROM test (Diacron International, Grosseto, Italy). This test determines hydroperoxides (breakdown products of lipids and other organic substrates generated by the oxidative attack of ROS), through their reaction with the chromogen N,N-diethylparaphenylenediamine. The results are expressed in arbitrary ‘Carratelli Units’ (CarrU), where 1 CarrU is equivalent to the oxidising power of 0.08 mg H_2_O_2_/dl.

### Global solar radiation

Information of the monthly solar radiation from Teagasc, Grange’s meteorological station (located on the research farm) was obtained from The Irish Meteorological Service, available at http://www.met.ie/climate/available-data/monthly-data.

### Statistical analysis

Of the 48 calves enrolled, 1 calf from group Ctl-In died at 5 months of age due to a pneumonia. Additionally, calves with missing values were removed from the analysis. Therefore, 3 animals were removed for the 25OHD analysis (n = 44) and 1 for the haematology data (n = 46).

All statistical analysis were performed in RStudio (version 4.0.3). After assessing that the ANOVA assumptions were met a within-between subject design was used for analysing the effects of treatment, sunlight, and time and its interactions on the 25OHD serum levels. Individual calves were included as a random effect and weight difference as a covariate. Differences between treatments were tested by pairwise comparison with Bonferroni correction. This analysis was done using *tidyverse, rstatix,* and *psych* packages.

A multiple factor analysis (MFA) was performed on the cell counts for neutrophils (Neu), eosinophils (Eos), basophils (Bas), monocytes (Mon), and lymphocytes (Lym) collected from the haematology analysis. The haematology profile was done over 8 time points through the curse of the trial. Therefore, the cell counts (× 10^3^ μl/ml) for each cell type from all the time points was gather in one group. Thus, the group “Neu” was formed with the neutrophil cell counts from the 8 sampling points, and the same procedure was done with each cell. Then, MFA was done with 5 groups of cells (Neu, Mon, Bas, Eos and Lym), whereas treatment was used as categorical supplementary variable. The MFA was done with 46 individuals and 41 variables using the *FactoMineR* and *Factoextra* packages^55^.

The collective differences between groups in each cell type was further analysed by multivariate analysis of variance (MANOVA) using Pillai’s test. Data was assessed for normality, multicollinearity, and homogeneity of variance. A statistically significant difference on the combined dependent variable was followed by Welch’s ANOVA test, pairwise comparisons were done by Games–Howell test. Eosinophil data was analysed by Kruskal–Wallis test followed by Dunn’s Test. All statistical tests were interpreted using a 5% level of significance. This analysis was done using *tidyverse, rstatix,* and *psych* packages. All the figures were produced using ggplot2 in RStudio.

## Supplementary Information

Supplementary Information.

## Data Availability

The datasets analysed during the current study are available from the corresponding author on reasonable request.
