# Supplementary material for: Low serum vitamin D concentrations in Spring-born dairy calves are associated with elevated peripheral leukocytes
Source: Sci Rep. 2021 Sep 23;11:18969. doi: 10.1038/s41598-021-98343-8 (PMC8460825; doi:10.1038/s41598-021-98343-8)
Supplement: Supplementary file 1 — Supplementary Information. [file 41598_2021_98343_MOESM1_ESM.docx]

**Supplemental. Figure 1. Correlation circle from MFA analysis of the cell profile observed between groups**. The haematology profile for all time points was analysed by MFA as described in material and methods section. The correlation circle shows the relationship between variables and how they are represented based in the quality of its representation (cos2=0.2). Lym, Bas and Mon are well represented in Dim1, whereas Neu, Eos are represented in Dim2. Ctl-In (n=11), VitD-In (n=12), Ctl-Out (n=12) and VitD-Out (n=11).


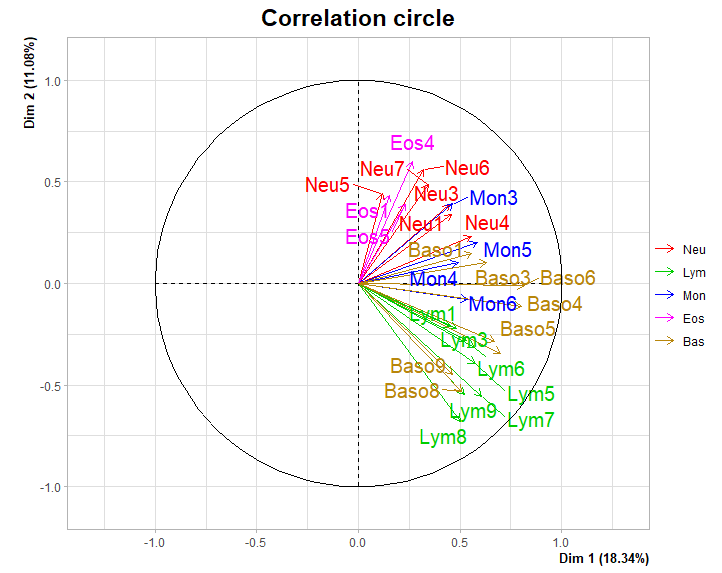


**Supplemental. Figure 2. Collective differences in the cell profile within each group at all time points.** Data shows the boxplot of WBC, neutrophils, eosinophils, basophils, lymphocytes, and monocytes within each group at all time points**.** Collective differences between groups were analysed by MANOVA as described in material and methods section. Pink area shows references values Merck Veterinary Manual. Dots represent outlier values. * *P* < 0.05. Ctl-In (n=11), VitD-In (n=12), Ctl-Out (n=11) and VitD-Out (n=10).


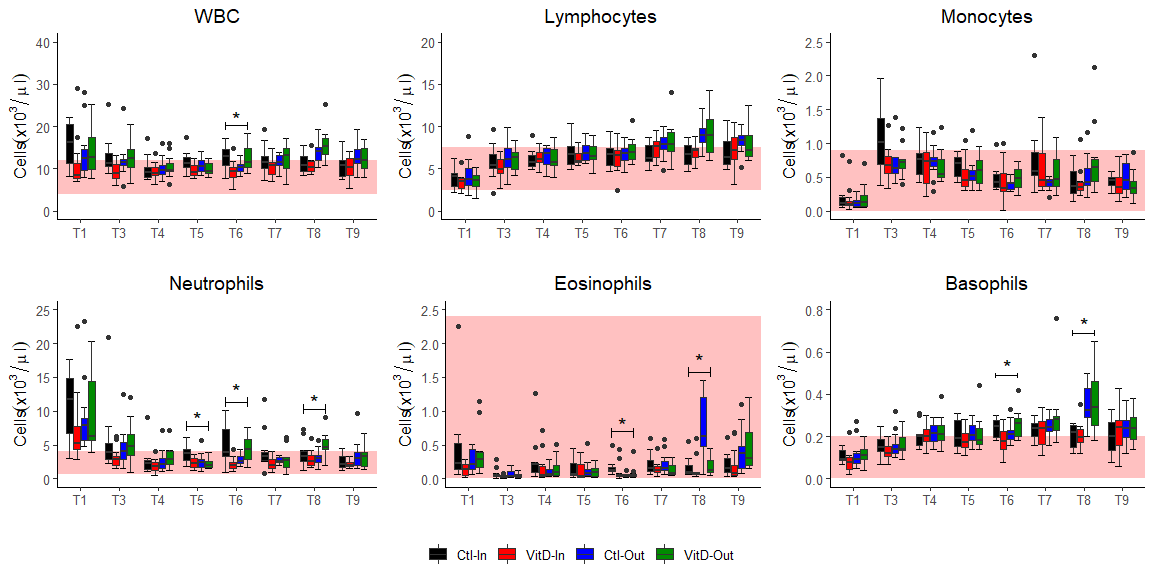


**Supplemental Table 1**

| **Supplemental Table 1. Multiple Factor Analysis results for within individual inertia of the groups^1^** | | |
| --- | --- | --- |
|  | **Dim.1** | **Dim.2** |
| Vit D-Out | 2.295542309 | 1.380713741 |
| Ctl-Out | 2.417169815 | 1.014944399 |
| Vit D-In | 2.832678364 | 7.508729728 |
| Ctl-In | 3.813751515 | 5.328866525 |

^1^Within individual inertia measures the heterogeneity of the groups along each dimension. The groups with similar values are closely to each other for that dimension.
